# Lipidomic Analysis of Hand Skin Surface Lipids Reveals Smoking-Related Skin Changes

**DOI:** 10.3390/metabo13020254

**Published:** 2023-02-09

**Authors:** Tian Chen, Mengzhen Zhao, Zhenxing Mao

**Affiliations:** 1 Division of Public Health Service and Safety Assessment, Shanghai Municipal Center for Disease Control and Prevention, Shanghai 200336, China; 2 NMPA Key Laboratory for Monitoring and Evaluation of Cosmetics, Shanghai 200336, China; 3 College of Public Health, Zhengzhou University, Zhengzhou 450001, China

**Keywords:** smoking, skin surface lipids, sphingolipids, glycerolipids, sterol lipids

## Abstract

Smoking contributes to the formation of skin wrinkles and reduces skin function, but the mechanism is not yet fully proven. This study aims to compare and analyze the effects of smoking on skin lipids and to further investigate the harmful effects of smoking on the skin. A total of 40 subjects (20 male smokers and 20 healthy control males) were recruited for this study. Measurement of hand skin-surface lipids (SSLs) in smoking and healthy control groups was undertaken using ultra-performance liquid chromatography quadrupole time-of-flight mass spectrometry (UPLC-Q-TOF-MS). Multivariate data analysis was used to investigate the differences in SSLs between the two groups. There were 1230 lipids detected in the two groups and significant differences in SSLs’ composition were observed between them. Under selected conditions, 26 types of lipid with significant differences were observed between the two groups (*p* < 0.05). Sphingolipids (SP) and glycerolipids (GL) were significantly increased, and sterol lipids (ST) were significantly reduced. Smoking causes changes in skin lipids that disrupt skin homeostasis, making the skin more fragile and more susceptible to skin aging and diseases.

## 1. Introduction

The skin is the primary barrier against external infections and environmental toxins, and it serves as a barrier between the body and the external environment [1]. It is composed of the epidermis, the dermis, and the hypodermis [2]. The epidermis is the skin’s natural protective barrier that provides structural support and limits the entry of chemicals. It consists of four layers: the stratum corneum, the stratum granulosum, the stratum spinosum, and the stratum basale [3]. Importantly, the distribution of skin lipids in the stratum corneum affects the skin barrier function, which limits water loss from within the body and entry of exogenous chemicals from the external environment [4]. Skin surface lipids (SSLs) are composed of sebocyte-, keratinocyte-, and microbe-derived lipids. The SSLs play a significant role in regulating skin conditions through physicochemical, biochemical, and microecological mechanisms [5]. They are divided into eight categories, including fatty acids (FA), glycerolipids (GL), glycerophospholipids (GP), sphingolipids (SP), sterol lipids (STs), prenol lipids (PRs), saccharolipids (SLs), and polyketides (PKs) [2]. Studies have shown that the composition and content of SSLs play a key role in the normal physiological function of the skin barrier and therefore alter the skin’s condition [6]. Ceramides, also known as Cers, play an important part in the skin’s capacity to structure and maintain its barrier function against the passage of water [7], and alterations in their levels can disrupt skin homeostasis leading to various skin disorders, such as atopic dermatitis [8,9]. In addition, studies have shown that the composition and content of DGs and STs are associated with acne and aging of the skin [10,11]. A change in the composition of SSLs can disrupt or impair the function of the skin barrier, which can result in a variety of skin diseases [12,13,14]. SSLs are susceptible to a variety of factors that have been extensively studied, such as gender, age, diet, environmental factors, etc. [15,16,17]. However, there are few reports of studies on the connection between SSLs and behavioral habits.

Smoking cigarettes is a significant problem for the health of the general population, which is associated with the pathogenesis of a variety of clinical disorders [18]. According to WHO reports, cigarette smoking kills more than eight million people every year around the world [17]. It is known that smoking is a risk factor for pulmonary diseases, including chronic obstructive pulmonary disease (COPD), asthma, and pulmonary fibrosis [19,20]. The skin is the first tissue that comes into contact with cigarette smoke, which affects the skin through external and internal exposure [21]. Several studies have shown that smoking is a factor in both the formation of wrinkles and the decline in skin function [22]. Additionally, smoking is also an important modifiable risk factor that increases the likelihood and severity of skin disease development [23]. The negative consequences that smoking has on skin health and disease have been widely established, but the influence that smoking has on skin lipids is still not fully understood [24,25].

Skin lipidomic analysis provides an approach for studying lipids by quantifying the changes in individual lipids that identify differences in lipid composition, which provides support for studies on the involvement of lipids in the health and disease of the skin [26]. In this study, ultra-performance liquid chromatography quadrupole time-of-flight mass spectrometry (UPLC-Q-TOF-MS), a powerful analytical method, was used to investigate SSLs’ variations under smoking conditions. This study analyzes and compares the differences in hand SSLs between male smokers and healthy control males, as well as the effects of smoking on lipid metabolism. The aim was to provide a theoretical basis for the development of skin management and smoking cessation.

## 2. Materials and Methods

### 2.1. Chemicals and Reagents

Formic Acid (FA), Acetonitrile (AN), Methanol (MT), N-propanol (NPA), Isopropanol (IPA), Ammonium Formate—all solvents were LC grade. Vortex Mixers, Mass Spectrometer (Waters UPLC-VION QTOF MS, Framingham, MA, USA), Nitrogen evaporator.

### 2.2. Study Population

A total of 40 subjects (20 male smokers and 20 controls, aged 45–59 years) in the Henan area were selected for this study. The smoking group was made up of chronically exposed individuals who had been smoking cigarettes with filters for more than 10 years, and the control group was non-smokers with little or no exposure to the tobacco environment. All volunteers were healthy and had no previous history of common skin diseases, such as contact dermatitis, eczema, or urticaria. In addition, the criteria for inclusion required that participants should not use any skin-care products, such as hand cream, for 3 days before participating in the experiment. Volunteers who were unable to complete the entire experiment or who had sensitive skin were excluded from the study. All of the volunteers were given full disclosure of the objectives of the study, and they all signed informed consent forms. This study was registered in the China Clinical Trial Center and approved by the Ethics Committee of Zhengzhou University, approval number (ID: ChiCTR2000034103).

### 2.3. Sample Collection and Storage

The volunteers were instructed to refrain from using any cleansers or other skincare products for a period of three days prior to the study. Volunteers washed their hands with clean water and sat for 30 min at a temperature of 20 °C and a relative humidity of 40–60% before sample collection. The experimenter placed a Corneofix^®^ test strip on the tiger’s mouth of each volunteer’s right hand (which is located on the back of the hand at the junction of the thumb and index finger, between the first and second metacarpal bones, as shown in Figure S1), removed the strip after 30 min, and placed it in a 2 mL EP tube that was then stored at –80 °C.

### 2.4. Sample Treatment

After removing the samples from freezers maintained at –80 °C, 1.5 mL of the reagent mixture (consisting of chloroform and methanol) was added. The mixture was vortexed and incubated for 1 hour at room temperature. Then, the Corneofix^®^ test strips were removed from the EP tubes, and the nitrogen evaporator was utilized to blow-dry the leftover lipid-containing extract. Finally, 200 µL of IPA/ACN/distilled water (65:30:5) was used for reconstitution, and the sample was transferred to the vial for detection after vortexing. During the analysis, we controlled the analytical performance using a quality control sample, which was prepared by mixing sample extracts. One QC sample was inserted for every eight samples analyzed.

### 2.5. Analysis Conditions

#### 2.5.1. Liquid Chromatography Conditions

The chromatographic column was a Waters ACQUITY UPLC CSH C18 Column, 1.7 μm, 2.1 mm × 100 mm. The flow rate was maintained at 0.3 mL/min. The injection volume was 3.0 μL, and the column was thermostated at 50 °C. Mobile phase A was 60% water + 40% ACN  +  10 mM ammonium formate + 0.1% formic acid and mobile phase B was 10% ACN  +  90% IPA  +  0.1% formic acid. During UPLC runs, the injector needle was washed with the mobile phase. Table S1 (Supplementary Materials) shows mobile phase A and B gradient elution settings.

#### 2.5.2. Mass Spectrometry Conditions

Electrospray ionization (ESI) was carried out with positive ion mode acquisition and a mass scan range of 50–1200 m/z. The data collection method was MS^E^ mode. The source and desolation temperatures were kept at 120 °C and 500 °C, respectively. The capillary voltage was set at 3.0 kV and the desolvation gas flow was 900 L/h. The detailed ion source conditions are shown in Table S2.

### 2.6. Multivariate Data Analysis and Statistical Analysis

Multivariate data analysis was performed by Waters Progenesis QI 3.0.3 (Waters Corporation) and Ezinfo 3.0.3 (Waters Corporation). Firstly, the collected raw data were inputted into the QI software for peak alignment processing. Secondly, the samples were divided into smoking and control groups for peak extraction. The compound information obtained by peak picking was then imported into Ezinfo 3.0.3. Orthogonal Partial Least Squares Discriminant Analysis (OPLS-DA) was carried out for the purpose of identifying the factors that were most responsible for discrimination, with the following selection criteria: variable influence on projection (VIP) > 1, *p* < 0.05, fold change (FC) > 2. Finally, the differentiated lipids between the two groups were imported into MetaboAnalyst 4.0 (https://dev.metaboanalyst.ca/, accessed on 17 January 2023), and the metabolite set enrichment analysis (MSEA) algorithm from Lipid Maps in MetaboAnalyst was used to explore the lipid metabolism pathways enriched by the screened differential lipids.

Statistical significance was calculated with the Student’s *t*-test using IBM SPSS Statistics 21.0 (IBM, Armonk, NY, USA); a statistical probability of *p* < 0.05 was considered significant. For all analyses, *** *p* < 0.001; ** *p* < 0.01; * *p* < 0.05.

## 3. Results

### 3.1. Variations in the Main Class of Lipids

**Figure 1:**
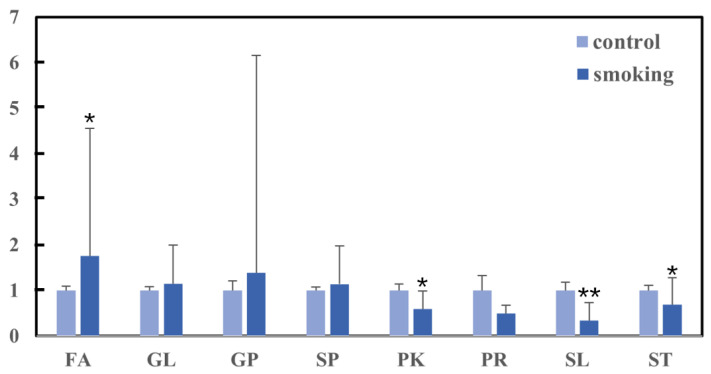
The relative amounts of the eight major lipids in the smoking and control groups. These units are ‘relative to the value for non-smokers, which is set at a value of 1’. ** *p* < 0.01; * *p* < 0.05.

This study identified 1230 lipids and separated them into eight major classes. The relative abundance of each major class in the smoking and control groups was computed. The relative amounts of the eight primary lipid classes that were found in each of the two groups is displayed in Figure 1, which illustrates the distinctions that exist between the two groups. In the smoking group, there was a significant reduction in three of the main classes (PKs, SLs, and STs) and an increase in FAs (*p* < 0.05). The remaining four classes did not show any significant changes. The results showed there was a difference between the smoking and control groups.

### 3.2. OPLS-DA Analysis of Hand Lipids from the Smoking Group and the Control Group

**Figure 2:**
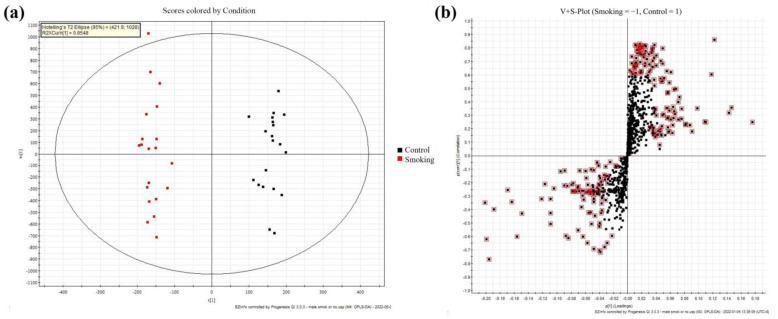
(**a**) Orthogonal projections to latent structures discriminant analysis (OPLS-DA) score plot of SSLs’ composition between smoking and control groups. (**b**) S-plot of selected components of differences in skin surface lipids (SSLs) between smoking and control groups.

SSLs from the smoking and control groups were analyzed using untargeted lipidomic methods based on the fine stability of UPLC-Q-TOF-MS. The OPLS-DA model was used for multivariate data analysis. The resulting model score plot is shown in Figure 2a. These results demonstrated good separation and significant differences between the smoking and control SSLs’ samples. With the S-plots and with VIP > 1 as a condition, differences between the smoking and control groups were further selected, as shown in Figure 2b.

### 3.3. Identification of Important Individual Lipids in the Smoking and Control Groups

Multivariate data analysis indicated that there was a significant difference between the smoking and control groups in the composition of SSLs. Therefore, it was required to combine the OPLS-DA models in order to discover the most significant differential lipid. With VIP > 1, *p* < 0.05, and fold change (FC) > 2 as screening conditions, 26 lipid components with significant differences were eventually identified between the two groups, as shown in Table 1. From Table 1, it can be seen that the differential sphingolipids in the two groups were mainly ceramide, glucose ceramide (GlcCer), and galactosylceramide (GalCer). Similarly, the glycerolipids were primarily composed of diacylglycerol (DG) and triglycerides (TGs), with levels of both glycerolipids being higher in the smoking group.

**Figure 3:**
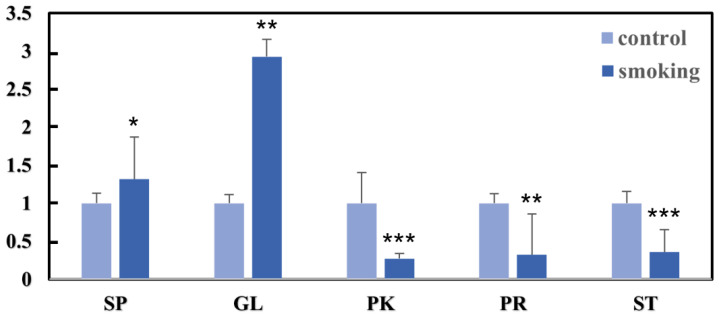
Comparison of differences in lipids in the smoking and control groups. These units are ‘relative to the value for non-smokers, which is set at a value of 1’. *** *p* < 0.001; ** *p* < 0.01; * *p* < 0.05.

**Figure 4:**
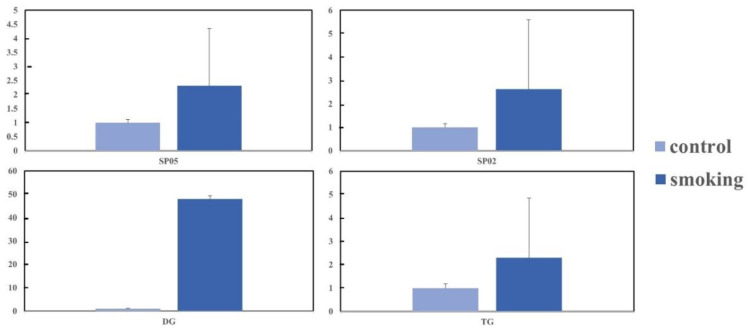
Comparison of differences in sphingolipids and glyceroglycolipids between the smoking and control groups. These units are ‘relative to the value for non-smokers, which is set at a value of 1’.

The relative abundance of differential lipids was calculated and is shown in Figure 3; a total of 26 significantly different lipids were divided into five main classes of lipids. Compared with the control study, levels of SPs and GLs were significantly higher in the smoking group, whereas the three main classes (PKs, PRs, and STs) had lower levels. Additionally, when the relative changes in subclasses of SPs and GLs were analyzed (Figure 4), the levels of neutral glycosphingolipids (SP05), ceramides (SP02), DG, and TGs were significantly higher in the smoking group.

### 3.4. Enrichment Analysis of Skin Lipid Metabolism between Smoking and Control Groups

**Figure 5:**
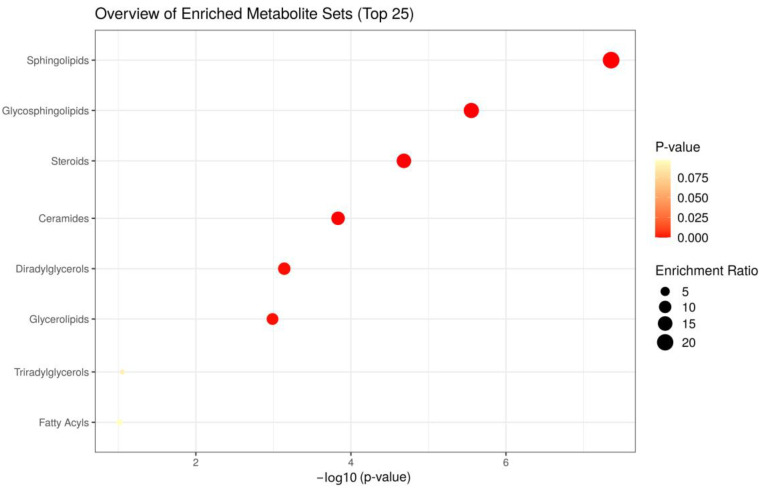
Metabolic pathway maps of important individual lipids responsible for differentiating between the smoking and control groups.

Enrichment analysis of SSLs between the smoking and control groups revealed significantly different lipid metabolism. Figure 5 shows that the enrichment analysis module identified eight metabolic pathways associated with skin conditions during smoking, including sphingolipid metabolism, glycosphingolipid metabolism, sterol metabolism, ceramide metabolism, diglyceride metabolism, glycerolipid metabolism, triglyceride metabolism, and fatty acid metabolism. The circle for sphingolipid metabolism is the largest of these lipid metabolisms, and is located in the upper right-hand corner of the picture. This particular lipid metabolism is thought to be the one that is most damaged by smoking.

## 4. Discussion

This study was conducted to confirm the relationship between changes in skin lipids caused by smoking and the health of the skin. Cigarette smoke causes a series of skin problems, such as skin wrinkling and aging, loss of skin elasticity, and impaired skin barrier homeostasis; it is also associated with psoriasis, acne, and skin cancers [27]. In this study, a total of 1230 different lipids were identified, and among the eight primary classes of lipids, the relative abundances of FAs were found to be significantly increased in the smoking group, whereas the abundances of PKs, SLs, and STs were significantly decreased. Based on VIP > 1, *p* < 0.05, and FC > 2, 26 individual lipid species that contributed significantly to the group differences were selected, and SPs were predominant among these lipids. The discovered SPs’ metabolic pathway contained two lipids, glycosphingolipids (GSLs) and Cers, both of which contributed to the differences between groups.

### 4.1. Analysis of Differences in Sphingolipid

The metabolism of SPs plays a significant role in the maintenance of the skin barrier and regulates cellular processes [14], which are related to the proliferation and differentiation of keratinocytes and to the function of the skin barrier [28]. Studies have shown that many skin diseases, such as psoriasis, are associated with abnormal SPs’ metabolism. The significance of SPs as structural and signaling molecules and their role in inflammation as factors contributing to vascular endothelial abnormalities in the development of psoriasis is well documented [14]. Alterations in SPs’ metabolism affect epidermal skin-lipid composition and skin barrier function. The screened differential lipids Cer and GSL are also involved in the metabolism of SPs in this study.

Cers, the main lipid component of the stratum corneum, play an important role in epidermal barrier maintenance, epidermal self-renewal, and immune modulation [29]. In the stratum corneum, they prevent the loss of water and the penetration of harmful chemicals from the environment [7]. The results of this study showed that there was a significant difference in the Cers content between the smoking group and the control group, with the Cers content in the smoking group being significantly higher than that in the control group. Studies have shown that the skin homeostasis of smokers is disrupted due to the effects of tobacco smoke, which contains both stable and unstable free radicals and reactive oxygen species (ROS) [30]. Acid sphingomyelinase (ASM) is a lysosomal protein that is activated by reactive oxygen species in smoke and catalyzes the conversion of sphingomyelin into ceramide. ASM contributes to membrane-ceramide accumulation [30,31], and it is also associated with the increased expression of inflammatory cytokines and matrix metalloproteinase 9 [32]. In addition, oxidative stress induced by cigarette smoke activates neutral sphingomyelinase (nSMase) 2—the only sphingomyelinase—which increases ceramide production to affect apoptosis. According to these results, Cers are important in maintaining epidermal homeostasis, but excessive levels may result in irreversible cell death. Inappropriate apoptosis impairs keratinocyte proliferation and differentiation, which can result in dysfunction of the epidermal permeability barrier [33]. The results suggest that substances such as reactive oxygen species in cigarette smoke disrupt skin barrier homeostasis by altering the content of Cers in the skin.

GSLs are important for maintaining the integrity of the membrane and provide unique recognition sites; they are also involved in a variety of human diseases, including autoimmune diseases, ichthyosis, and cancer [34,35,36]. This study found that there were significant differences in the levels of GalCers and GlcCers in the smoking group compared with the healthy control group, and there were higher levels of both GSLs in the smoking group. The mechanism of smoking’s effects on GSLs is unknown. However, there is a close relationship between GSLs and the skin barrier. As an activator of natural killer (NK) cells, GalCers initiate an immune response and activate an inflammatory cascade, which is significantly elevated in the lesional skin of Hidradenitis Suppurativa (HS) patients [37]. GlcCers are critical for the formation of the skin permeability barrier, which can release Cers in the skin [38]. Accumulation of GlcCers in the stratum corneum causes immature lipid sheets to be formed, which decrease the barrier function. Holleran et al. suggested that the persistence of GlcCers may be the primary cause of the membrane structural abnormalities leading to the skin lesions in type 2 Gaucher disease [39]. Our results suggest that the skin barrier function of the hands of smokers is lower than that of non-smokers, and is associated with altered GLS content.

### 4.2. Analysis of Differences in Glycerolipids

The most abundant component of human sebum are TGs, which is composed of three chains of fatty acids esterified to glycerol. It provides a control mechanism for the penetration of water through the skin surface—a crucial function of the skin’s ability to regulate moisture—which TGs accomplish by playing an important role in this function [40]. The results of this study showed that there were significant differences in TGs’ levels between the smoking group and the healthy control group. In the smoking group, the TGs were presented at higher levels. On one hand, the elevated TGs’ content is associated with the activation of sterol regulatory element binding-protein (SREBP-1) by cigarette smoke, which induces the expression of genes involved in TGs’ production to increase TGs’ synthesis [41,42]. On the other hand, free radicals in tobacco smoke cause oxidative damage to lipids, leading to lipid disorders and ultimately increasing TGs’ concentrations [43]. Importantly, studies have shown that the accumulation of triglycerides in SC can affect the barrier function and lead to abnormal skin permeability, thus stimulating epidermal proliferation [44], which can cause dry skin [45], ichthyosis, atopic dermatitis, and other skin diseases [46]. In addition, TGs indirectly impact on skin conditions by affecting the content of DGs and FAs, which causes the abnormal differentiation of the epidermis by inducing an influx of calcium ions into keratinocytes [47]. Based on these results, we think that higher levels of TGs caused by smoking may have more effects on how skin diseases start and worsen.

DGs are precursors for TGs’ synthesis, which is recognized as a lipid molecule with a signaling function [48]. As a result of this study, the relative content of TGs in the smoking group was higher than that in the healthy control group. In addition, the nicotine found in tobacco smoke has the effect of activating genes involved in glycerol metabolism, such as Lin3, Pnpla2, and Pnpla7. These genes are responsible for catalyzing the conversion of phosphatidic acid into DGs [49]. DGs are considered to be an influential factor in regulating skin melanin production [50]. The increase in DGs activates the protein kinase C (PKC) pathway, which is involved in the proliferation and differentiation of melanocytes [51,52]. It is thought that DGs activate melanogenesis by a PKC-dependent pathway [53]. From these results, it can be inferred that increased DGs can alter the skin color of smokers. Therefore, we inferred that the deepening of skin color in smokers is related to the increase in DGs.

### 4.3. Analysis of Differences in Sterol Lipids and Polyketides

STs play important functional roles in mammalian biology and have the ability to dynamically regulate the fluidity of cell membranes. They are also a gene expression regulator in lipid metabolism, influencing cholesterol transport and storage [54]. It is known that STs can be oxidized to produce oxysterols, which play key roles in RedOx homeostasis, inflammatory status, lipid metabolism, and induction of cell death [55]. In the smoking group, a significant reduction in STs’ levels was observed compared with that of the healthy control group (Figure 4). Free radicals in cigarette smoke induce lipid peroxidation [56], which accelerates the oxidation of STs to oxysterols. A lower level of STs is associated with cigarette smoke-induced lipid peroxidation in the smoker. Several studies have shown that oxysterols affect a number of processes associated with aging, including inflammation, oxidative stress, cell death and survival, and epigenetics [57,58]. These results demonstrate a close relationship between cigarette smoke-induced skin aging and lipid homeostasis in STs and oxysterols.

PKs are derived from microorganisms, which have important anticancer, antimicrobial, antioxidant, and anti-inflammatory effects [59]. In a previous study, PKs were found to be significantly lower in the skin of children in the acne group [60]. In this study, a significant decrease in PKs was found in the skin of subjects in the smoking group compared with the control group. There is a lack of research on the mechanism of the effect of smoking on PKs, but changes in PKs’ levels suggest changes in the microflora of the skin surface, which may affect the microbial action of the skin. The mechanisms by which smoking reduces PKs and their effects on the skin should be further explored in future studies.

The main strength of this study is the analysis of the damage of smoking to the hand skin by lipidomics, which contributes to the understanding of the mechanisms associated with smoking-induced hand skin disorders. In addition, the requirements for volunteers during the experiment were very strict, which ensured the authenticity and reliability of the results. However, a limitation of this study is that it is a cross-sectional study that cannot specifically estimate skin damage, as this study compared the two groups of study populations to evaluate the effects of smoking on skin lipids.

In short, there is a strong connection between alterations in SSLs and the distinctive skin condition that smoking causes. This study may provide new insights into the management of smokers’ skin and provide new clues for skin researchers and dermatologists. Additionally, the results of this study further explain the harm caused by smoking to the human body, which increases the persuasion and action for smoking cessation.

## Figures and Tables

**Table 1:** Screened individual skin surface lipids (SSLs) considered most important for distinguishing between the smoking and control groups.

Class	Accepted Description	m/z	Anova (p)	q Value	Max Fold Change	Highest Mean
Sphingolipids	Cer(d18:0/28:0(28OH))	724.7194	0.001333	0.006793	2.530351	smoking
Sphingolipids	Cer(d18:2/30:0)	732.725	0.003913	0.017549	2.980895	smoking
Sphingolipids	Cer(d18:2/28:0(2OH))	720.6875	0.016101	0.052893	2.397683	smoking
Sphingolipids	Cer(d18:0/30:0(30OH))	752.751	5.17 × 10^−05^	0.000421	3.519314	smoking
Sphingolipids	GlcCer(d15:2(4E,6E)/18:0)	684.5442	1.18 × 10^−14^	7.67 × 10^−13^	Infinity	control
Sphingolipids	GlcCer(d14:2(4E,6E)/16:0)	642.4955	2.74 × 10^−07^	3.94 × 10^−06^	2.452004	control
Sphingolipids	GlcCer(d15:2(4E,6E)/22:0)	740.6035	9.04 × 10^−10^	2.25 × 10^−08^	3.328931	smoking
Sphingolipids	GlcCer(d18:2/22:0)	782.6508	6.06 × 10^−06^	6.58 × 10^−05^	3.283642	smoking
Sphingolipids	Galactosylceramide (d18:1/18:1(9Z))	726.5911	0	0	Infinity	control
Sphingolipids	Galactosylceramide (d18:1/26:1(17Z))	838.7142	8.22 × 10^−08^	1.37 × 10^−06^	3.35593	smoking
Sphingolipids	Sphing-6E-enine 4R-sulfate	413.2672	5.89 × 10^−05^	0.000475	2.987902	control
Glycerolipids	TGs(50:0)	852.8037	6.10 × 10^−05^	0.000487	3.870762	smoking
Glycerolipids	TGs(52:0)	880.8356	0.000234	0.001585	6.50787	smoking
Glycerolipids	TGs(58:5)	954.8499	0.043424	0.11684	5.250341	control
Glycerolipids	TGs(58:2)	960.8959	0.04401	0.118043	2.951669	control
Glycerolipids	DG(38:3)	647.5596	1.40 × 10^−13^	7.44 × 10^−12^	437.4144	smoking
Glycerolipids	DG(36:3)	619.5285	7.41 × 10^−12^	2.99 × 10^−10^	72.23256	smoking
Glycerolipids	DG(34:3)	591.4976	0.001338	0.006793	13.26929	smoking
Sterol Lipids	Cyclopassifloic acid E	570.4028	8.61E-09	1.74 × 10^−07^	4.337149	control
Sterol Lipids	Acetylpinnasterol	489.3218	1.49 × 10^−05^	0.000146	3.667711	control
Sterol Lipids	Echinasteroside C	630.4237	2.36 × 10^−05^	0.000219	3.118912	control
Sterol Lipids	12alpha-Hydroxy-3-oxo-5beta-cholan-24-oic Acid	391.2853	3.31 × 10^−05^	0.000296	4.530213	control
Polyketides	4′-Hydroxy-5,7-dimethoxy-8-methylflavan	301.1435	0	0	Infinity	control
Polyketides	Squamocin-V	640.5516	3.46 × 10^−06^	4.08 × 10^−05^	2.343713	control
Polyketides	Formononetin 7-O-(6″-acetylglcoside)	473.1465	9.89 × 10^−06^	0.000102	2.937326	control
Prenol Lipids	bacteriohopane-32,33,34-triol-35-cyclitolguanine	750.5594	0.006071	0.024858	3.100748	control

## Data Availability

All data generated or analyzed during this study are included in this article. Further enquiries can be directed to the corresponding author.

## Supplementary Materials

The following supporting information can be downloaded at: https://www.mdpi.com/article/10.3390/metabo13020254/s1, Table S1. The gradient elution conditions with mobile phase A and mobile phase B. Table S2. The detailed ion source conditions of QTOF-MS. Figure S1. Right hand tiger mouth position.
