# Supplementary material for: Lipidomic Analysis of Hand Skin Surface Lipids Reveals Smoking-Related Skin Changes
Source: Metabolites. 2023 Feb 9;13(2):254. doi: 10.3390/metabo13020254 (PMC9963340; doi:10.3390/metabo13020254)

Supplementary Materials

Table S1. The gradient elution conditions with mobile phase A and mobile phase B.

Time(min)	A phase (%)	B phase (%)
0.00	80	20
1.0	80	20
5.0	40	60
11.0	20	80
18.0	10	90
19.0	0	100
21.0	0	100
21.10	80	20
22.00	80	20

Table S2. The detailed ion source conditions of QTOF-MS.

Parameters	ESI+
Sample temperature	10.0℃
Column temperature	50.0℃
Analysis mode	sensitivity
Capillary voltage	3.0kV
Ion source temperature	120℃
Desolvation temperature	500℃
Cone gas flow	50L/h
Desolvation gas flow	900L/h

Figure S1. Right hand tiger mouth position.

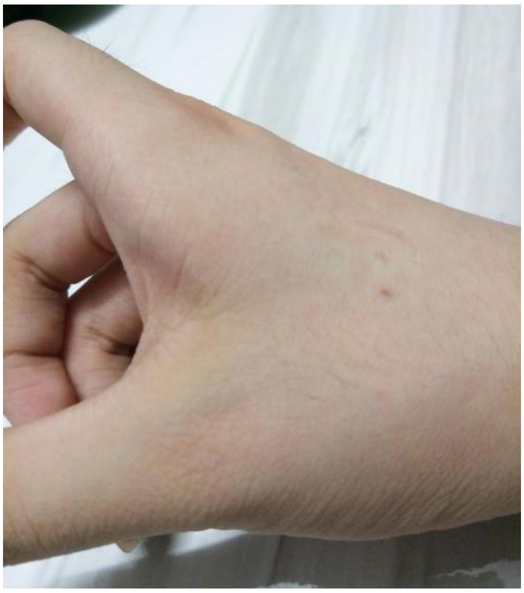

Supplement: Supplementary file 1 [file metabolites-13-00254-s001.zip › metabolites-2197229-SI.pdf]
